# Beneficial Effects of the Direct AMP-Kinase Activator PXL770 in In Vitro and In Vivo Models of X-Linked Adrenoleukodystrophy[Fn FN6]

**DOI:** 10.1124/jpet.122.001208

**Published:** 2022-08

**Authors:** Pierre-Axel Monternier, Parveen Parasar, Pierre Theurey, Pascale Gluais Dagorn, Navtej Kaur, Tavarekere N Nagaraja, Pascale Fouqueray, Sébastien Bolze, David E. Moller, Jaspreet Singh, Sophie Hallakou-Bozec

**Affiliations:** Poxel SA, Lyon, France (P.-A.M., P.T., P.G.D., P.F., S.B., D.E.M., S.H.-B.) and Departments of Neurology (P.P., N.K., J.S.) and Neurosurgery (T.N.N.), Henry Ford Health System, Detroit, Michigan

## Abstract

X-linked adrenoleukodystrophy (ALD) is a severe orphan disease caused by mutations in the peroxisomal *ABCD1* transporter gene, leading to toxic accumulation of Very Long-Chain Fatty Acids (VLCFA – in particular C26:0) resulting in inflammation, mitochondrial dysfunction and demyelination. AMP-activated protein kinase (AMPK) is downregulated in ALD, and its activation is implicated as a therapeutic target. PXL770 is the first direct allosteric AMPK activator with established clinical efficacy and tolerability. Methods: We investigated its effects in ALD patient-derived fibroblasts/lymphocytes and *Abcd1* KO mouse glial cells. Readouts included VLCFA levels, mitochondrial function and mRNA levels of proinflammatory genes and compensatory transporters (*ABCD2-3*). After PXL770 treatment in *Abcd1* KO mice, we assessed VLCFA levels in tissues, sciatic nerve axonal morphology by electronic microscopy and locomotor function by open-field/balance-beam tests. Results: In patients’ cells and *Abcd1* KO glial cells, PXL770 substantially decreased C26:0 levels (by ∼90%), improved mitochondrial respiration, reduced expression of multiple inflammatory genes and induced expression of *ABCD2-3*. In *Abcd1* KO mice, PXL770 treatment normalized VLCFA in plasma and significantly reduced elevated levels in brain (-25%) and spinal cord (-32%) versus untreated (*P* < 0.001). Abnormal sciatic nerve axonal morphology was also improved along with amelioration of locomotor function. Conclusion: Direct AMPK activation exerts beneficial effects on several hallmarks of pathology in multiple ALD models in vitro and in vivo, supporting clinical development of PXL770 for this disease. Further studies would be needed to overcome limitations including small sample size for some parameters, lack of additional in vivo biomarkers and incomplete pharmacokinetic characterization.

**SIGNIFICANCE STATEMENT:**

Adrenoleukodystrophy is a rare and debilitating condition with no approved therapies, caused by accumulation of very long-chain fatty acids. AMPK is downregulated in the disease and has been implicated as a potential therapeutic target. PXL770 is a novel clinical stage direct AMPK activator. In these studies, we used PXL770 to achieve preclinical validation of direct AMPK activation for this disease - based on correction of key biochemical and functional readouts in vitro and in vivo, thus supporting clinical development.

## Introduction

Adrenoleukodystrophy is a neurometabolic disease caused by mutations in the ATP Binding Cassette Subfamily D member 1 (*ABCD1*) gene, on the X chromosome (Kemp et al., 2012). Affecting approximately one in 5000–17,000 people (Bezman et al., 2001; Engelen et al., 2012; Wiens et al., 2019), ALD is the most common peroxisomal disease as well as the most common leukodystrophy (Schmidt et al., 2020).

Adrenomyeloneuropathy (AMN) is the most common clinical form of ALD, characterized by a progressive axonal degeneration/demyelination of the spinal cord and peripheral nerves, which affects nearly all males (Engelen et al., 2012; Wiesinger et al., 2015). Independently of AMN, 60% of male patients will also develop cerebral ALD (C-ALD); onset is often during childhood (∼40%) but it may also arise in adolescents/adults (∼20%). This form of ALD is characterized by inflammatory white matter brain demyelination and lesions (Berger et al., 2014). The X-linked recessive nature of ALD leads to a profound impact in males, with female carriers most often showing a less severe, later life onset of the symptoms (Engelen et al., 2012).

ABCD1 is required for import and oxidation of very long-chain fatty acids (VLCFA - ≥ 22 carbons) by peroxisomes. Its dysfunction causes VLCFA accumulation in plasma and in cells in all tissues including brain, adrenal cortex and spinal cord (Igarashi et al., 1976; Valianpour et al., 2003; Singh and Pujol, 2010; Engelen et al., 2012). ALD can be readily diagnosed by the presence of increased plasma VLCFA – C24:0 and, in particular C26:0, as well by an increase in the C26:0/C22:0 ratio (Rattay et al., 2020). Even though there is no clear association between disease severity and the degree of VLCFA accumulation in plasma (and fibroblasts from ALD patients), evidence suggests that VLCFA accumulation in normal-appearing white matter may correlate with clinical status (Asheuer et al., 2005). Downstream effects of VLCFA accumulation include ER stress, mitochondrial dysfunction, reactive oxygen species generation and apoptosis (Kemp et al., 2012, 2016; Turk et al., 2020). Importantly, a central role of neuroinflammation has been established, in particular in the development of demyelination in C-ALD (Singh and Pujol, 2010; Görtz et al., 2018), and a specific increase of proinflammatory cytokines even in asymptomatic patients (Marchetti et al., 2018).

AMPK is a crucial bioenergetic sensor. It is a heterotrimeric protein consisting of three distinct subunits (*α*, *β*, *γ*); there are 12 possible complexes since each subunit exists as two or three isoforms. AMPK activation has effects of potential importance in ALD: 1) inhibition of neural cell apoptosis (Liu et al., 2021), inflammation and fatty acid synthesis; and 2) enhancement of mitochondrial function, biogenesis and fatty acid oxidation (Steinberg and Carling, 2019). Importantly, we previously observed that AMPK is downregulated in postmortem brain white matter tissue from ALD patients as well as in patient-derived fibroblasts and lymphocytes; we also showed that deletion of AMPK in glial cells from *Abcd1* KO mice exacerbates their disease-related phenotype (Singh and Giri, 2014; Singh et al., 2015, 2016). These findings implicated AMPK as a specific therapeutic target in ALD (Weidling and Swerdlow, 2016). In ALD patient-derived cells, metformin - a weak and indirect AMPK activator - at a suprapharmacological concentration (5mM - (Wang et al., 2019)) was shown to decrease levels of VLCFA, improve mitochondrial function, reduce markers of inflammation and dose-dependently induce *ABCD2* expression in ALD lymphocytes (Singh et al., 2016).

PXL770 is a small molecule, direct allosteric AMPK activator (Gluais-Dagorn et al., 2022) currently in Phase 2 development for nonalcoholic steatohepatitis (NASH). PXL770 binds to the ADaM binding site of the protein, and shows similar potency for AMPK complexes that contain *α*1 versus *α*2 subunits, with higher (nM) potency for *β*1 subunit containing complexes versus lower (*μ*M) potency with *β*2 subunit containing complexes. High selectivity for AMPK was shown by screening against a panel of receptors, ion channels, and transporters (Eurofins Cerep, Celle-Lévescault, France). A single potential additional target was identified, with weak inhibition of CCK1 during a receptor binding assay (Gluais-Dagorn et al., 2022). Substantial preclinical and clinical efficacy was demonstrated on multiple metabolic disease related features in animals and humans with nonalcoholic fatty liver disease (NAFLD), Type 2 diabetes, obesity and insulin resistance (Cusi et al., 2021; Fouqueray et al., 2021; Gluais-Dagorn et al., 2022). Importantly, PXL770 is the first direct AMPK activator to have progressed to clinical trials for any disease. Here, we showed that direct activation of AMPK ameliorates biochemical and functional features of ALD.

## Materials and Methods

### In Vitro

#### Human-Derived Cells

AMN and C-ALD human patient-derived cell lines (along with a healthy control cell line) were obtained from the NIGMS Human Genetic Cell Repository at the Coriell Institute for Medical Research (www.coriell.org). Fibroblasts were primary cells obtained from individual male donors (control: GM03348, AMN: GM17819, C-ALD: GM04934). Lymphocytes were obtained from individual male donors (control: GM03798, C-ALD:GM04673) and immortalized. Cells were cultured in Dulbecco's Modified Eagle Medium (DMEM, fibroblasts) or RPMI-1640 (lymphocytes), supplemented with 15% (fibroblasts) or 10% (lymphocytes) fetal bovine serum (FBS, BioAbchem Inc #72-0400) and antibiotics (Penicillin, 100 units/ml; streptomycin, 100 *μ*g/ml; HyClone #SV30010), and maintained at 37°C in 5% CO2. All experiments were performed at passage 6 to 8.

#### *Abcd1*-Null Mouse-Derived Cells

Animals were handled as described in the in vivo experiments below. Primary astrocyte-enriched cultures were prepared from the whole cortex of either wild-type C57BL/6 or *Abcd1* KO 1/2-day-old pups (*n* = 5) as described earlier (Singh et al., 2009). After 10 days confluent mixed glial cultures were used for the experiments.

#### Compounds and Treatment

PXL770 was synthetized by Poxel SA. The chemical structure was previously published (Gluais-Dagorn et al., 2022), and the synthetic route is described in patent WO2020099678. PXL770 was dissolved in DMSO (Sigma; Cat#D2650). Controls were treated with DMSO vehicle. Metformin Hydrochloride (Sigma, #1115-70-4) was dissolved in DMEM (Cytiva; SH30243.01). The media was changed every day with fresh drug treatment.

#### mRNA Levels/RT-qPCR

0.5 million AMN/C-ALD fibroblasts or *Abcd1* KO glial cells/well were plated in 6-well plates. 0.5 million lymphocytes were cultured in 25cm^2^ flasks as suspension cultures. After 72 hours of treatment, mRNA was isolated using Qiagen RNAeasy kit (Qiagen, #74104) and cDNA prepared using Qiagen RT II cDNA kit (Qiagen, #330401). Real-time quantitative PCR was conducted using Bio-Rad CFX^96^ Real-Time PCR Detection System and iTaq Universal SYBR Green Supermix (Bio-Rad, Catalog#1725124). Results are expressed as ratio of the targeted gene over the housekeeping gene (RLP27 gene). Sequences of the primers used are listed (Supplemental Table 1).

#### VLCFA Content

300K AMN/C-ALD fibroblasts or *Abcd1* KO glial cells were plated in 100mm petri dishes (Fisherbrand, #431762). 300K lymphocytes were cultured in 25cm^2^ flasks as suspension cultures. Cells were treated with PXL770 for one week. At the end of treatment, cells were washed with ice-cold phosphate buffered saline and one million cells per sample were pelleted and processed as follows: samples are adjusted to a final volume of 0.5–1 ml with LC-MS grade water and spiked with 10 ng of Lignoceric acid-d4 as an internal standard. The sample is acidified to pH 3 to 4 with dilute hydrochloric acid and extracted with isooctane-ethyl acetate (9:1) three times with equal volume. The extract is dried under nitrogen and the residue is reconstituted in methanol-water-ammonium acetate (75:25: 10 mM). Aqueous sodium hydroxide is added to a final concentration of 1 M, and the mixture is incubated at 37°C in dark under nitrogen for 3H. The samples are then acidified, extracted, and reconstituted as described above. The reconstituted fatty acid extracts are subjected to HPLC on Targa C8 column (2x10 mm) using methanol-aqueous ammonium acetate (10 mM) solvent mixture. The column is eluted with a gradient of methanol (75 to 90%) over 8 minutes at a flow rate of 0.25 ml/min. The column eluent is directly introduced to mass analyzer (QTRAP5500) and monitored for fatty acids using published pseudo MRM method. Under these conditions, the VLCFA elute between 5 and 8 minutes. Each fatty acid is quantitated against the added internal standard.

#### Bioenergetics

0.5 million AMN/C-ALD fibroblasts or *Abcd1* KO glial cells/well were plated in 6-well plates. 16-18 hours post plating the cells were treated with PXL770. 72 hours after the treatment cells were harvested with a cell lifter and washed twice with bicarbonate-free DMEM prewarmed at 37°C. 100K cells were replated in 175 *μ*l of bicarbonate-free DMEM in XFe96 plates per well and preincubated at 37°C for 1 hour for degassing before starting the assay procedure. Oxygen consumption rate (OCR) was measured using Seahorse XFe96 Analyzer (Agilent). After baseline measurements (average of the three first measurements), OCR was measured after sequential addition of oligomycin (1 *μ*M), carbonyl cyanide-p-trifluoro methoxyphenylhydrazone (FCCP, 0.25 *μ*M for human cells and 0.5 *μ*M for mouse cells), and Rotenone/antimycin A (1 *μ*M).

#### AMPK Phosphorylation

Total AMPK and p-AMPK protein levels were measured by Western-blot (8 or 12% polyacrylamide gels) in AMN/C-ALD fibroblasts and in *Abcd1* KO glial cells, as described (Supplemental Material), using the following antibodies: AMPK*α*1/2 (T-AMPK, Cell Signaling, #2532) and p-AMPK (Cell Signaling, #2535).

#### Inflammatory Genes Expression

C-ALD patient's derived lymphocytes were cultured in 25cm^2^ tissue culture flasks. The cells were cultured in Serum-free DMEM for cytokine analysis. The cells were treated for 72 hours. *Abcd1* KO glial cells were cultured in serum-free media overnight followed by pretreatment with PXL770 or vehicle 2 hours; TNF*α* (10ng/ml) and IL1*β* (10ng/ml) were then added to the media for 70 hours resulting in a total drug exposure period of 72 hours. mRNA levels were measured as described above in *mRNA levels/RT-qPCR.*

### In Vivo Studies

#### Animals

C57BL/6J (Stock N°000664) and *Abcd1* KO (B6.129-Abcd1tm1Kan/J, Stock No: 003716, backcrossed to C57BL/6J background) mice were purchased from Jackson Laboratory (Bar Harbor, ME) and maintained at the Henry Ford Health System (HFHS) animal facility on a 12/12H light/dark cycle with standard rodent chow and water ad libitum. All animal procedures were approved by the HFHS Animal Review Committee (IACUC#1050), and all animals received human care in compliance with the HFHS experimental guidelines and the National Research Council’s criteria for humane care (Guide for Care and Use of Laboratory Animals).

#### Compounds and Treatment

PXL770 (synthesized as noted above) was suspended in 0.5% CMC/Tween80 (Sigma, #C4888, #P1754), and was administered twice a day by oral gavage at 75 mg/kg. Control animals received vehicle (0.5% Carboxy MethylCellulose - CMC) administered once a day.

#### Pharmacokinetic (PK) Study

PXL770 was orally administered as a single dose of 75 mg/kg to 6–8-week-old *Abcd1* KO male mice. Blood was sampled at the following time points (3 animals per time point) after PXL770 administration: 0.15, 0.5, 1, 3, and 8 hours. Plasma was harvested and analyzed by LC/MS-MS for quantification of PXL770. The analytical work, including sample preparation and quantitative bioanalysis by LC/MS-MS was performed at Charles River laboratories. PK analysis was performed using a version of Phoenix WinNonlin (version 8.2).

#### VLCFA Content Measurements in 6–8-Week-Old *Abcd1* KO Mice

6–8-week-old *Abcd1* KO male mice were treated by twice daily oral gavage with PXL770 or vehicle for 8 weeks. At the end of treatment, mice were sacrificed with CO2 followed by harvesting of plasma and brain samples that were then snap frozen in liquid N_2_. Samples were then stored at -80°C until processed for VLCFA analysis as described in the in vitro section above.

#### Axonal Morphology, Behavioral Assessment, and Spinal Cord VLCFA Measurements in 13-Month-Old *Abcd1* KO Mice

Treatments with PXL770 or vehicle of 3 months in duration were initiated in 13-month-old *Abcd1* KO male mice. Axonal morphology, behavior, and spinal cord VLCFA content were assessed for all groups. For spinal cord *VLCFA* content measurements, a similar procedure as used for 6–8-week-old mice was employed, as described above.

#### Microscopy Analysis

Animals were prepared as described (Supplemental Material). Briefly, deeply anesthetized mice were perfused with fixative (2.5% glutardialdehyde in a 4% solution of paraformaldehyde in PBS) and dissected to expose the gluteus bilaterally. The sciatic nerves were located using previously developed methods and stored in the fixative solution. The sciatic nerve tissues were postfixed in 1% osmium tetroxide, processed through grades of ethanol and embedded in araldite. They were cut into 1 *μ*m thick transverse sections using an ultramicrotome and stained with toluidine blue to visualize and quantify myelinated fibers. Myelin and morphometric electronic microscopy (EM) studies were performed on thin sections (1*μ*M) of sciatic nerve. Criteria used for blinded evaluation were as follows: 1) appearance of axons: regular, nearly circular bundles or stellate (starlike) appearance and 2) hypermyelination: in-folding of myelin sheath (Pujol et al., 2002).

#### Behavioral Tests

The Balance Beam Test was performed the week before tissue harvesting. Mice underwent four acclimatization trials (one trial per day) and one final test measurement as follows: a 3-inch-wide plank was placed across the open space (space was secured to protect the animals in case of fall). The start side was very brightly lit, and the end side was dark, and ended in a hiding place with treats after the animals were allowed to cross the beam. The blinded observer was facing the back of the animal – primarily looking only the back paws slip. In some cases, the animals never consistently crossed the beam from one side to the other. In this case, the beam was divided into segments, and the number of slips and time were scored to cross a certain number of segments. A scoring system was used to assess the ability of the animals to traverse the beams as described in Table 1.

**TABLE 1:** Balance Beam Test scoring system

Beam Balance Tests	
0	Balances with steady posture
1	Grasps side of beam
2	Hugs the beam and one limb falls down from the beam
3	Hugs the beam and two limbs fall down from the beam
4	Attempts to balance on the beam but falls off (>20 sec)
5	Attempts to balance on the beam but falls off (>10 sec)
6	Falls off: No attempt to balance or hang on to the beam (<10 sec)

For the Open Field Test, the mice were acclimatized to the test chamber for 60 minutes every day for 4 consecutive days, and for 30 minutes before data acquisition during the data collection days. All data collection was performed in an undisturbed environment, preferably in the mornings at the same time of the day, for an hour a day, for 4 consecutive days. The animals were placed in the center of the activity-field arena, which is a transparent plexiglass cage (W x D x H; 260 × 260 × 400 mm) equipped with two photobeam sensor rings to register horizontal and vertical activity. Testing lasted 30 minutes.

#### Sample Size Determination and Statistics

For in vitro experiments with patient-derived cells, the number of replicates chosen (3–5 for VLCFA and 4–6 for additional parameters) in each experiment was based on our prior experience and knowledge of the degree of variability in the tested parameters and known mean differences between healthy and diseased cells (Singh and Giri, 2014; Singh et al., 2015, 2016). A smaller number of replicates was chosen for mouse glial cells as this cell type was employed as a confirmatory step prior to in vivo testing. For in vivo experiments, mouse sample size calculations were performed assuming a power of 0.8 to detect a potential 50% improvement in VLCFA levels and a significance level of *P* < 0.05 (with *n* = 10 per group, we expected a detectable effect size of 1.3 SD and a 95% CI half-width of 0.88 SD); In the initial mouse study, *n* = 12–15 was chosen to account for any mortality during the long treatment plan. In 13-month-old mice, a sample size of 8 per group was selected based on results obtained in younger mice and limited availability of older mice. Data were analyzed using Graph Pad prism software. Statistical analysis was conducted on raw values including all groups. Normality was tested using the Shapiro-Wilk test. Model characterization and significance of the treatment effect were tested using one-way ANOVA followed by post hoc Dunnett’s multiple comparison test when data were normally distributed, and Kruskal-Wallis followed by post hoc Dunn’s multiple comparison test when data were not normally distributed, versus untreated AMN/C-ALD and Abcd1 KO cells/mice.”

## Results

### PXL770 Normalizes VLCFA Levels in C-ALD and AMN Fibroblasts and Lymphocytes

**Fig. 1.**
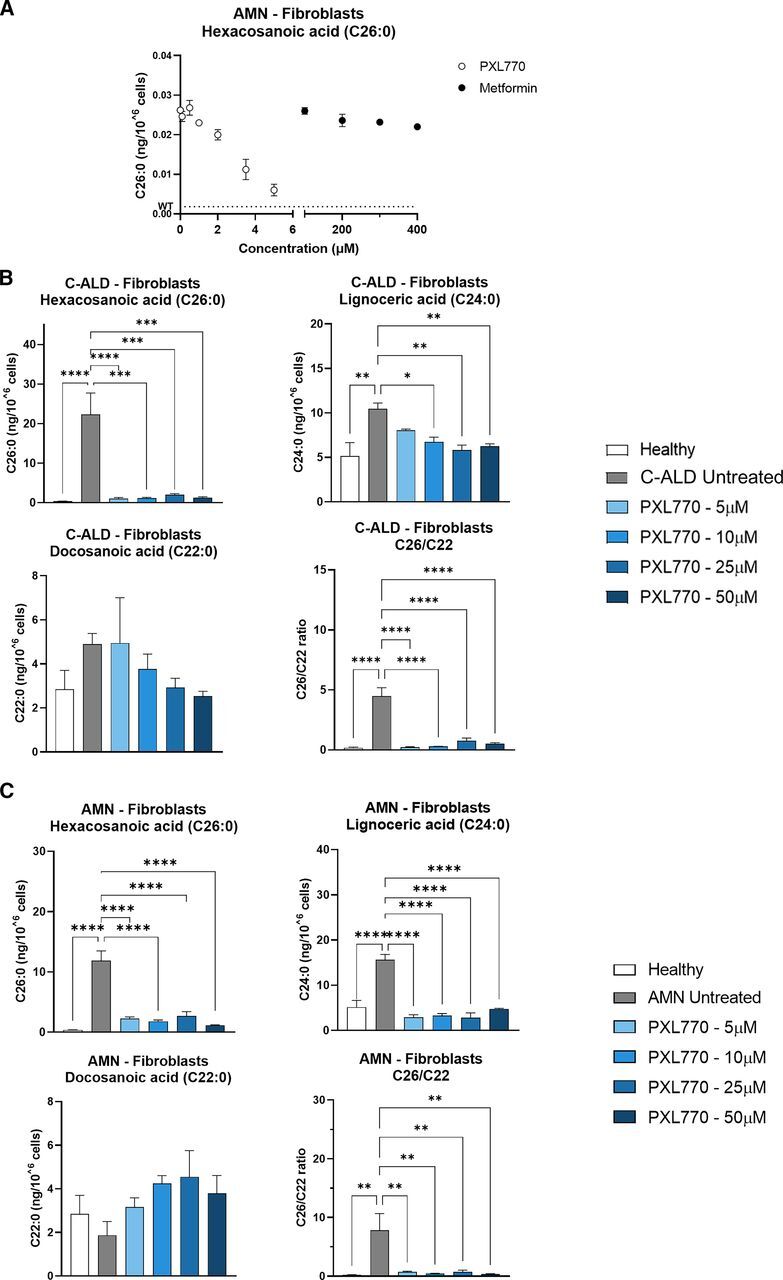
PXL770 treatment improves VLCFA levels in C-ALD and AMN patient-derived fibroblasts. Fibroblasts were isolated from individual patients with AMN (A) and C-ALD (B, C). (A) AMN fibroblasts were exposed for 7 days to PXL770 at 0.1, 0.5, 1, 2, 3.5, 5, 10, 25, and 50 *μ*M or to Metformin at 100, 200, 300, and 400 *μ*M. VLCFA levels were analyzed by LC-MS after extraction of total lipids from pelleted cells. Fibroblasts from a healthy donor, a C-ALD patient (B) or an AMN patient (C) were exposed to control media or the indicated concentrations of PXL770 followed by LC-MS measurements of specific lipids and the ratio of C26:0/C22:0 VLCFA as described for (A). Data are mean ± S.E.M. *n* = 5 replicates for (A), 3 for (B), and 5 for (C). Similar results were obtained in two additional independent experiments with these same patient donor cell lines. *P ≤ 0.05, **P ≤ 0.01, ***P ≤ 0.001, ****P ≤ 0.0001 versus C-ALD/AMN untreated (Dunnett’s test).

We investigated the effect of PXL770 on VLCFA levels in patient-derived cells after 7 days of treatment. Given that a high (5 mM - suprapharmacological (Wang et al., 2019)) Metformin concentration, an indirect AMPK activator, was previously reported to reduce VLCFA levels in ALD cells (Singh et al., 2016), we sought to compare the effects of PXL770 on C26:0 levels in AMN fibroblasts at different concentrations (0.1, 0.5, 1, 2, 3.5 and 5 *μ*M) to Metformin at clinically relevant levels (LaMoia and Shulman, 2021) (100, 200, 300, and 400 *μ*M). PXL770 reduced C26:0 with an IC50 of 3.1 *μ*M (95% CI 2.3 – 4.3 *μ*M), whereas Metformin had no observable effect (Fig. 1A).

After these results, and given that no clear maximal inhibition was observed at 5 *μ*M, additional experiments were conducted with PXL770 at 5, 10, 25 and 50 *μ*M in two independent sets of experiments conducted with one cell line obtained from an adult male ALD patient (with AMN - “AMN fibroblasts”) and another cell line from a younger male ALD patient (with C-ALD - “C-ALD fibroblasts”). Fibroblasts from a healthy male donor were used as a control.

Fibroblasts isolated from the C-ALD patient displayed C26:0 levels 60-fold higher compared with healthy control (*P* < 0.0001, Fig. 1B). PXL770 at any given concentration strongly reduced the C26:0 level compared with untreated cells (-91 to -95%, *P* < 0.001, Fig. 1B). A similar trend with lower magnitude was observed with C24:0, with an increase in patient’s cells of 2-fold compared with healthy control (*P* < 0.01, Fig. 1A), and a significant reduction induced by PXL770 at 10, 25 and 50 *μ*M (-36% *P* < 0.05, -44% and -40% *P* < 0.01, respectively, versus untreated, Fig. 1B). Consistent with previous reports (Moser et al., 1981), the levels of C22:0 were not significantly affected in C-ALD fibroblasts compared with healthy control, and no significant change was observed in PXL770-treated versus untreated cells. Importantly, the C26:0/C22:0 ratio, established as a robust marker for ALD diagnosis (Valianpour et al., 2003; Rattay et al., 2020), increased by 26.5-fold in patient-derived cells (*P* < 0.0001 versus healthy, Fig. 1B). PXL770 treatment substantially reduced this ratio (-83 to -95%, *P* < 0.0001 versus untreated, Fig. 1B).

Similar VLCFA variations were observed in AMN patient-derived fibroblasts, with C26:0 levels increased by 32-fold compared with healthy control (*P* < 0.0001, Fig. 1C), and an important effect of PXL770 to decrease mean levels compared with untreated cells (-77 to -90%, *P* < 0.0001, Fig. 1C). C24:0 levels were also increased by 3-fold compared with healthy control (*P* < 0.0001, Fig. 1C), and PXL770 incubation restored levels down to control (*P* < 0.0001 versus untreated, Fig. 1C). Similar to C-ALD patient-derived fibroblasts, C22:0 levels remained stable over the conditions studied and the elevated C26:0/C22:0 ratio was reduced by 91 to 96% upon PXL770 treatment compared with untreated cells (*P* < 0.01, Fig. 1C).

Interestingly, the results obtained in fibroblasts were also reproduced in C-ALD and AMN patient-derived lymphocytes – with respect to C26:0, C24:0 and C26:0/C22:0 levels (Supplemental Fig. 1, A and B).

These data indicated that treatment with PXL770 (5-50 *μ*M) nearly normalizes elevated VLCFA levels in cells derived from C-ALD and AMN patients.

Of note, these effects were paralleled by an increase in AMPK phosphorylation (Supplemental Fig. 2).

### PXL770 Improves Mitochondrial Function and Induces Compensatory Transporter Genes in C-ALD and AMN Patients’ Fibroblasts and Lymphocytes

**Fig. 2.**
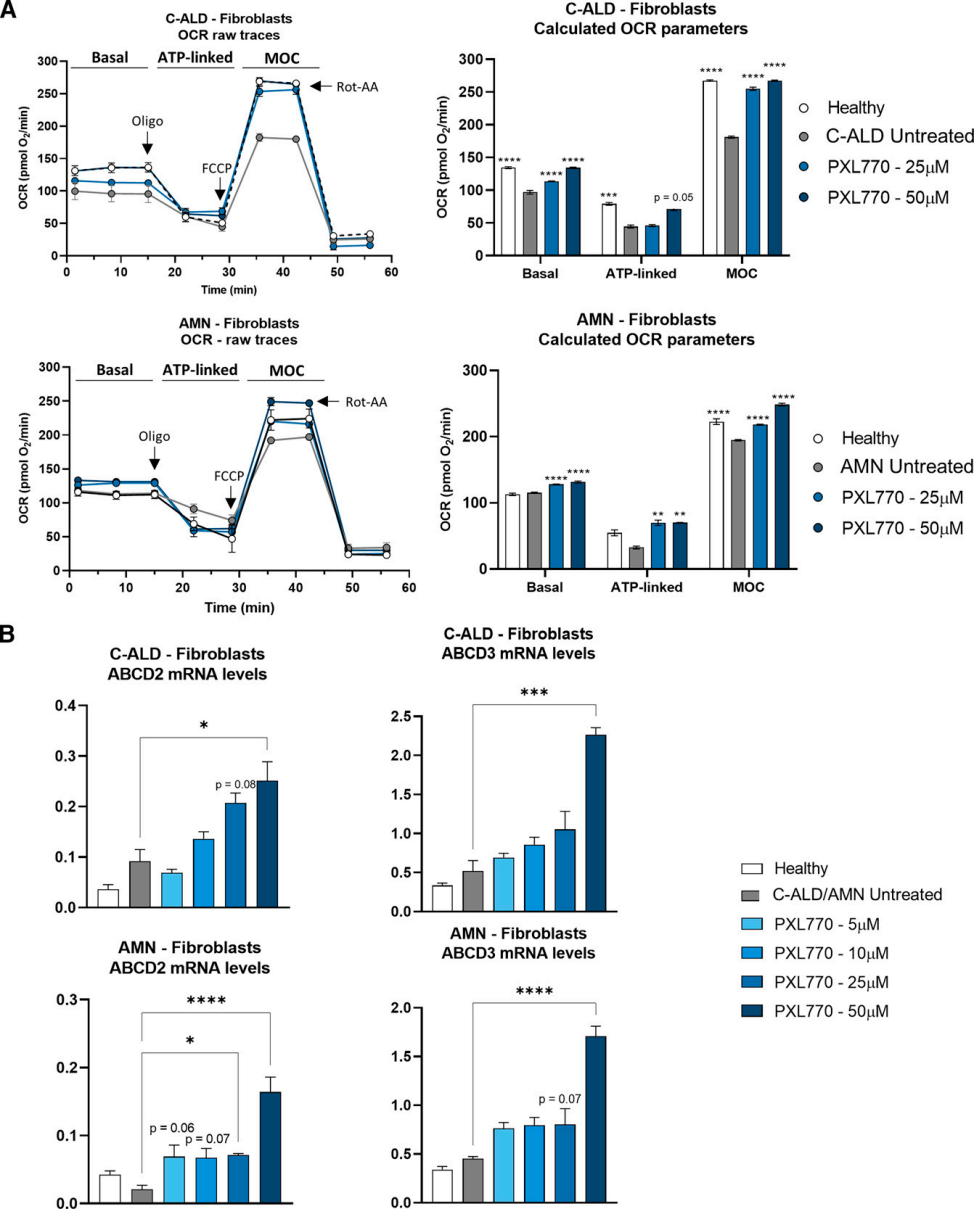
PXL770 mediated improvement of VLCFA levels is associated with restored mitochondrial function and increased compensatory gene expression in C-ALD and AMN patients’ fibroblasts. Fibroblasts were isolated from C-ALD and AMN patients and exposed for 72 hours to PXL770 at 5, 10, 25 or 50*μ*M. (A) OCR was measured using a Seahorse Analyzer and bioenergetic parameters were evaluated by sequential additions of: oligomycin (Oligo - 1 *μ*M), FCCP (0.25 *μ*M) and Rotenone-Antimycin A (Rot-AA - 1 *μ*M). Basal = first three measurements, ATP-linked = OCR drop after oligo addition, MOC = OCR after addition of FCCP. Data are mean ± SEM *n* = 6 replicates per patient cell line. (B) ABCD2 and 3 mRNA levels evaluated by RT-qPCR, normalized by RLP27 expression (no unit). Data are mean ± SEM *n* = 4-6 replicates per patient cell line. *P ≤ 0.05, **P > 0.01, ***P < 0.001, ****P < 0.0001 (Dunnett’s test versus untreated).

Beyond VLCFA accumulation, mitochondrial dysfunction is a major cellular feature of ALD. Thus, we sought to determine if the PXL770 effects on VLCFA in fibroblasts was associated with mitochondrial function improvements. For this purpose, we measured the OCR in intact living cells using a Seahorse XF Analyzer. Mitochondrial function was altered in C-ALD cells compared with healthy control, as indicated by the reduced basal (-27%, *P* < 0.0001 versus healthy, Fig. 2A) and ATP-linked respiration (-44%, *P* < 0.001 versus healthy, Fig. 2A), as well as lower maximal oxidative capacity (MOC, -32%, *P* < 0.0001 versus healthy, Fig. 2A). PXL770 treatment rescued basal (+17% at 25 *μ*M and +38% at 50 *μ*M, *P* < 0.0001 versus untreated, Fig. 2A) and ATP linked respiration (+61% at 50 *μ*M, *P* = 0.05 versus untreated, Fig. 2A) as well as MOC (+40% at 25 *μ*M and + 47% at 50 *μ*M, *P* < 0.0001 versus untreated, Fig. 2A).

Even though basal respiration was not altered in AMN patient’s fibroblasts compared with healthy control, PXL770 treatment resulted in a significant increase in this parameter compared with untreated cells (+11%, *P* < 0.0001, Fig. 2A). Compared with healthy control, ATP linked respiration was reduced by 40% in AMN cells but without reaching significance (Fig. 2A), and MOC was significantly reduced by 12% compared with healthy control (*P* < 0.0001, Fig. 2A). PXL770 incubation at 25 and 50 *μ*M increased ATP linked respiration equally by 112% at both concentrations (*P* < 0.01 versus untreated, Fig. 2A), whereas MOC was increased by 11 and 27%, respectively, compared with untreated AMN cells (*P* < 0.0001, Fig. 2A). These results indicate that PXL770 mediated AMPK activation also improved the disease associated phenotype of C-ALD and AMN cells by improving mitochondrial function.

Upregulation of other functionally redundant peroxisomal ABC transporters, ABCD2 and ABCD3, has been suggested as a potential rescue mechanism for VLCFA import and oxidation (Kemp et al., 1998; Netik et al., 1999). To assess whether VLCFA reductions could potentially be driven by changes involving ABCD2 and ABCD3, mRNA levels corresponding to each transporter were measured in C-ALD and AMN fibroblasts and healthy control cells. In C-ALD fibroblasts, PXL770 treatment tended to upregulate ABCD2 expression at 25 *μ*M (2.3-fold, *P* = 0.08 versus untreated, Fig. 2B) and reached significance at 50 *μ*M (2.7-fold, *P* < 0.05 versus untreated, Fig. 2B), and upregulated ABCD3 at 50 *μ*M (4.4-fold, *P* < 0.001 versus untreated, Fig. 2B). In AMN fibroblasts, PXL770 tended to increase ABCD2 expression at 5 and 10 *μ*M (∼3-fold, *P* = 0.06-0.07 versus untreated, Fig. 2B) - the effect became significant at 25 and 50 *μ*M (3.4-fold and 7.8-fold, *P* < 0.05 and *P* < 0.0001 versus untreated, respectively, Fig. 2B), and ABCD3 at 25 *μ*M (1.8-fold, *P* = 0.07 versus untreated, Fig. 2B) and 50 *μ*M (3.8-fold, *P* < 0.0001 versus untreated, Fig. 2B). Of note, similar results were obtained in AMN patient’s lymphocytes (Supplemental Fig. 1C).

These results suggest that part of the positive effects of PXL770 on VLCFA in C-ALD and AMN cells may be mediated – at least in part - by increases in the expression of genes encoding compensatory ABCD2 and ABCD3 transporters.

### PXL770 Improves VLCFA Levels and Mitochondrial Function and Increases Compensatory Transporters in Glial Cells from *Abcd1* KO Mice

**Fig. 3.**
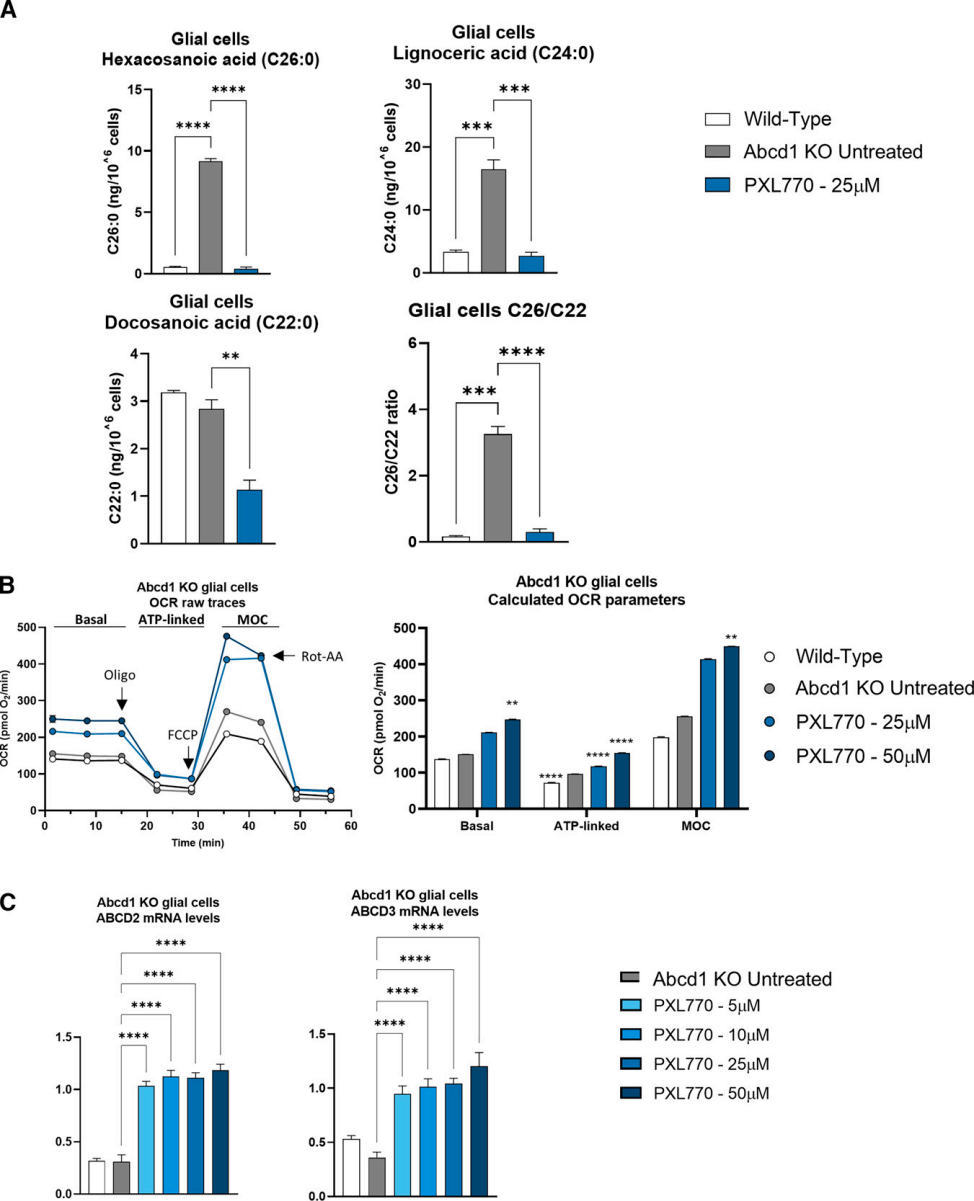
PXL770 treatment improves VLCFA levels, restores mitochondrial function and increases compensatory gene expression in Abcd1 KO mouse glial cells. Mixed glial cells (astrocyte-enriched) were isolated from 1-day-old wt and *Abcd1* KO mice. 10 days after culture cells were exposed to PXL770 at 5, 10, 25 or 50 *μ*M. (A) Cells were exposed for 7 days to the treatment and VLCFA levels were analyzed by LC-MS after extraction of total lipids from pelleted cells. Data are mean ± S.E.M., *n* = 2 replicates for wild-type and *n* = 3 replicates for *Abcd1* KO and PXL770. (B) Cells were exposed for 72 hours to the treatment and the OCR was measured using a Seahorse Analyzer. Bioenergetics parameters were evaluated by sequential additions of: oligomycin (1 *μ*M), FCCP (0.5 *μ*M) and Rotenone-Antimycin A (1 *μ*M). Basal = first three measurements, ATP-linked = OCR drop after oligo addition, Maximal Oxidative capacity = after addition of FCCP. Data are mean ± SEM, *n* = 6 replicates. (C) Cells were exposed for 72 hours to the treatment and mRNA levels were evaluated by RT-qPCR, normalized by Rlp27 expression (no unit). Data are mean ± SEM, *n* = 3–5 replicates. *P ≤ 0.05, **P ≤ 0.01, ***P ≤ 0.001, ****P ≤ 0.0001 (Dunnett’s test versus untreated).

*Abcd1* KO in mice is a validated disease model for ALD (ALD mice); these mice manifest VLCFA increases that are similar to levels observed in patients (McGuinness et al., 2003; Pujol et al., 2004); in older mice, a late onset neurologic phenotype is also evident (Pujol et al., 2002). A role for astrocytes, oligodendrocytes and microglia has been established in the pathophysiology of the disease - including sensitivity of glial cells to VLCFA toxicity (Berger et al., 2014; Gong et al., 2017; Görtz et al., 2018). Therefore, we investigated VLCFA levels, mitochondrial function and *Abcd2* and *Abcd3* mRNA expression in mixed glial cells culture from male *Abcd1* KO mice, exposed in vitro for 7 days to PXL770 (25 and 50 *μ*M). In line with the variations observed in C-ALD and AMN patient’s fibroblasts, *Abcd1* KO glial cells exhibited an increase of C24:0 (4.9-fold, *P* < 0.001, Fig. 3A) and C26:0 (17.5-fold, *P* < 0.0001, Fig. 3A), and no variation in C22:0 levels, compared with wild type (wt) mouse-derived cells. The C26:0/C22:0 ratio was increased by 19.7-fold compared with wt (*P* < 0.001, Fig. 3A). 25 *μ*M of PXL770 restored C26:0 and C24:0 levels to normal wt levels in ALD mice glial cells (*P* < 0.001 versus untreated, Fig. 3A). The C26:0/C22:0 ratio was diminished down to wt levels as well (*P* < 0.001 versus untreated, Fig. 3A), despite a significant reduction in C22:0 upon PXL770 treatment compared with untreated ALD mice glial cells (-60%, *P* < 0.01, Fig. 3A).

In glial cells isolated from ALD mice, basal respiration or MOC were apparently not impaired, and intriguingly ATP linked respiration was significantly increased by 32% compared with wt (*P* < 0.0001, Fig. 3B). Nevertheless, PXL770 treatment had substantial effects by increasing: basal respiration by 63% compared with untreated ALD mice glial cells at 50*μ*M (*P* < 0.01, Fig. 3B), ATP-linking oxygen consumption at 25 and 50 *μ*M by 21 and 58% (*P* < 0.0001 versus untreated, Fig. 3B), respectively, as well as MOC by 75% at 50 *μ*M (*P* < 0.01 versus untreated, Fig. 3B).

Interestingly, upregulation of mRNAs encoding the murine alternative transporters, Abcd2 and Abcd3, was also observed upon PXL770 treatment at all tested concentrations compared with untreated glial cells (2.6- to 3.8-fold, *P* < 0.0001, Fig. 3C).

Taken together, effects of PXL770 seen in patient cells were largely confirmed in ALD mouse glial cells – including normalization of VLCFA levels, stimulation of mitochondrial oxidative phosphorylation parameters, as well as upregulation of *Abcd2* and *Abcd3* mRNAs.

### PXL770 Treatment Improves Neuroinflammatory Gene Expression Markers in Patient-Derived Lymphocytes and in *Abcd1* KO Mouse Glial Cells

Given the importance of inflammation in the pathophysiology of ALD (Marchetti et al., 2018), we investigated the effects of 72 hours exposure to PXL770 at 10 *μ*M on the inflammatory profile in C-ALD patient’s lymphocytes, and in *Abcd1* KO glial cells stimulated with TNF*α* and IL1*β* (*Abcd1* KO TI).

**Fig. 4.**
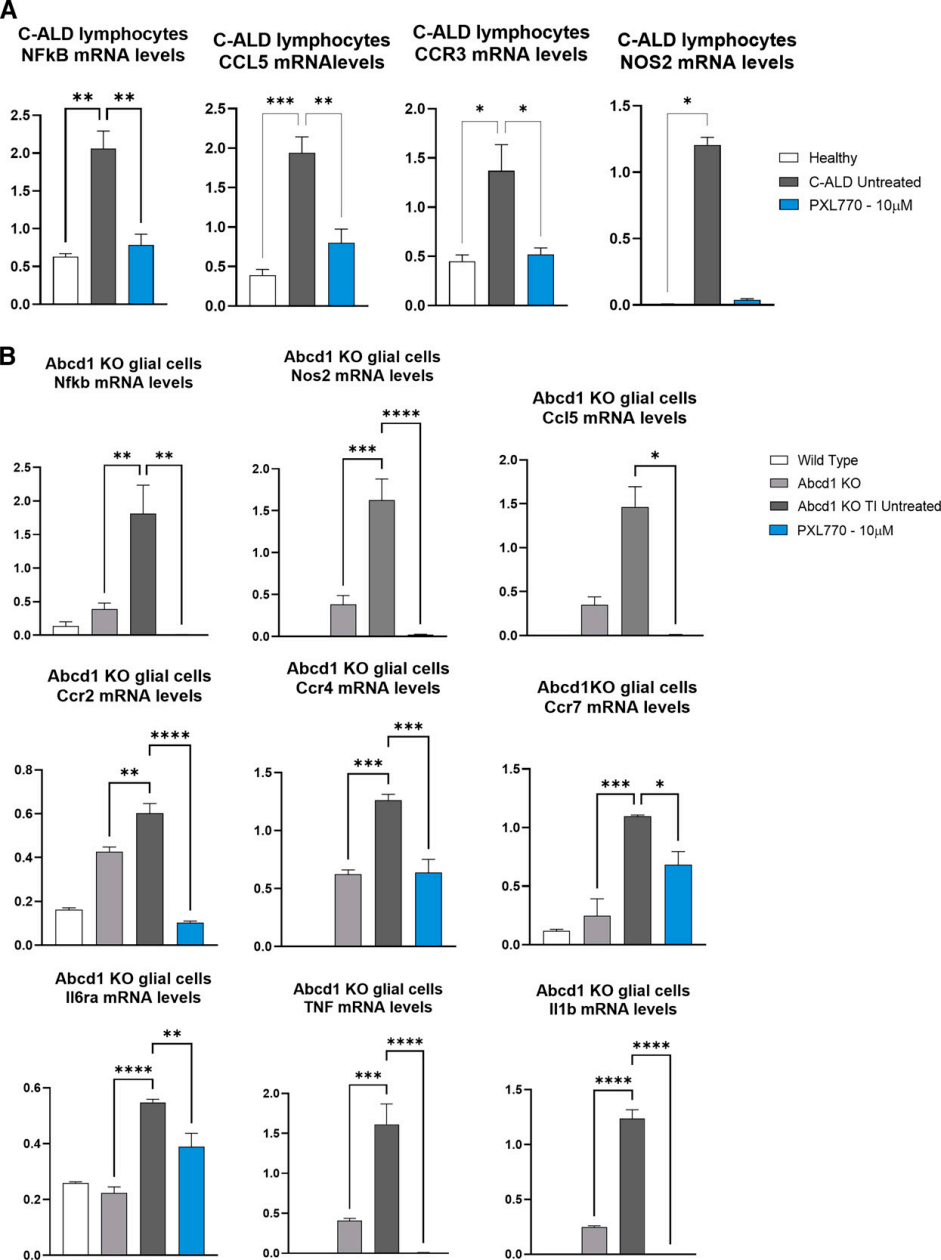
PXL770 treatment improves neuroinflammation gene expression markers in lymphocytes from a C-ALD patient and in Abcd1 KO mouse glial cells. (A) Lymphocytes were isolated from a C-ALD patient and (B) Mixed glial cells (astrocyte-enriched) were isolated from 1-day-old wt and *Abcd1* KO mice and matured for 10 days, then stimulated (TI) with TNF*α* (10ng/ml) and IL1*β* (10ng/ml) for 70 hours (during PXL770 treatment). Cells were exposed to PXL770 10 *μ*M for 72 hours and mRNA levels were evaluated by RT-qPCR, normalized by RLP27/Rlp27 expression (no unit). Data are mean ± S.E.M., *n* = 3 replicates. *P ≤ 0.05, **P ≤ 0.01, ***P ≤ 0.001, ****P ≤ 0.0001 (Dunnett’s or Kruskal Wallis test versus TI untreated – test wt versus TI untreated not shown).

In C-ALD lymphocytes, *NFKB*, *CCL5* and *CCR3* were identified as markers of interest given their increase in C-ALD cells versus healthy (3.1- to 5-fold, *P* < 0.05-0.001) and their significant decrease by PXL770 treatment versus untreated (∼ -60%, *P* < 0.05-0.01, Fig. 4A). *NOS2* was the most affected gene, increased by 168-fold in C-ALD lymphocytes compared with healthy cells (*P* < 0.05, Fig. 4A) whereas repressed by 97% by PXL770 (Kruskal-Wallis test not significant for *n* = 3, Fig. 4A).

In *Abcd1* KO TI mice glial cells, *Nfkb*, *Nos2*, *Ccl5*, *Ccr2*, *Ccr4*, *Ccr7*, *Il6ra*, *Tnfα* and *Il1b* were identified as markers of interest given their increase in *Abcd1* KO TI versus *Abcd1* KO (1.4- to 5-fold, *P* < 0.05-0.0001 [*Ccl5* not significant]) and their significant decrease by PXL770 treatment versus untreated (-29 to -100%, *P* < 0.05-0.0001, Fig. 4B).

Collectively, these results suggest that PXL770 treatment can have beneficial effects to ameliorate the proinflammatory phenotype of ALD diseased cells, in line with the well-known anti-inflammatory properties of AMPK activation.

### Plasma PK Parameters After PXL770 Administration in *Abcd1* KO Mice

6–8-week-old male *Abcd1* KO mice were dosed orally once with PXL770 at 75 mg/kg and plasma was sampled for PK analysis (*n* = 3 animals/time point). This dose has been previously established as well tolerated and optimal for systemic AMPK activation in other murine disease models (Gluais-Dagorn et al., 2022). The following parameters were obtained from the measured plasma concentration: t_max_ (time of first occurrence of maximum concentration) was 0.15 hour; C_max_ (maximum concentration) was 56.5 *μ*g/ml; and AUC_last_ (area under the curve from 0 to the last measurable concentration) was 81.5 *μ*g.h/ml.

### In Vivo PXL770 Treatment Decreases Plasma and Tissue VLCFA Levels in *Abcd1* KO Mice

**Fig. 5.**
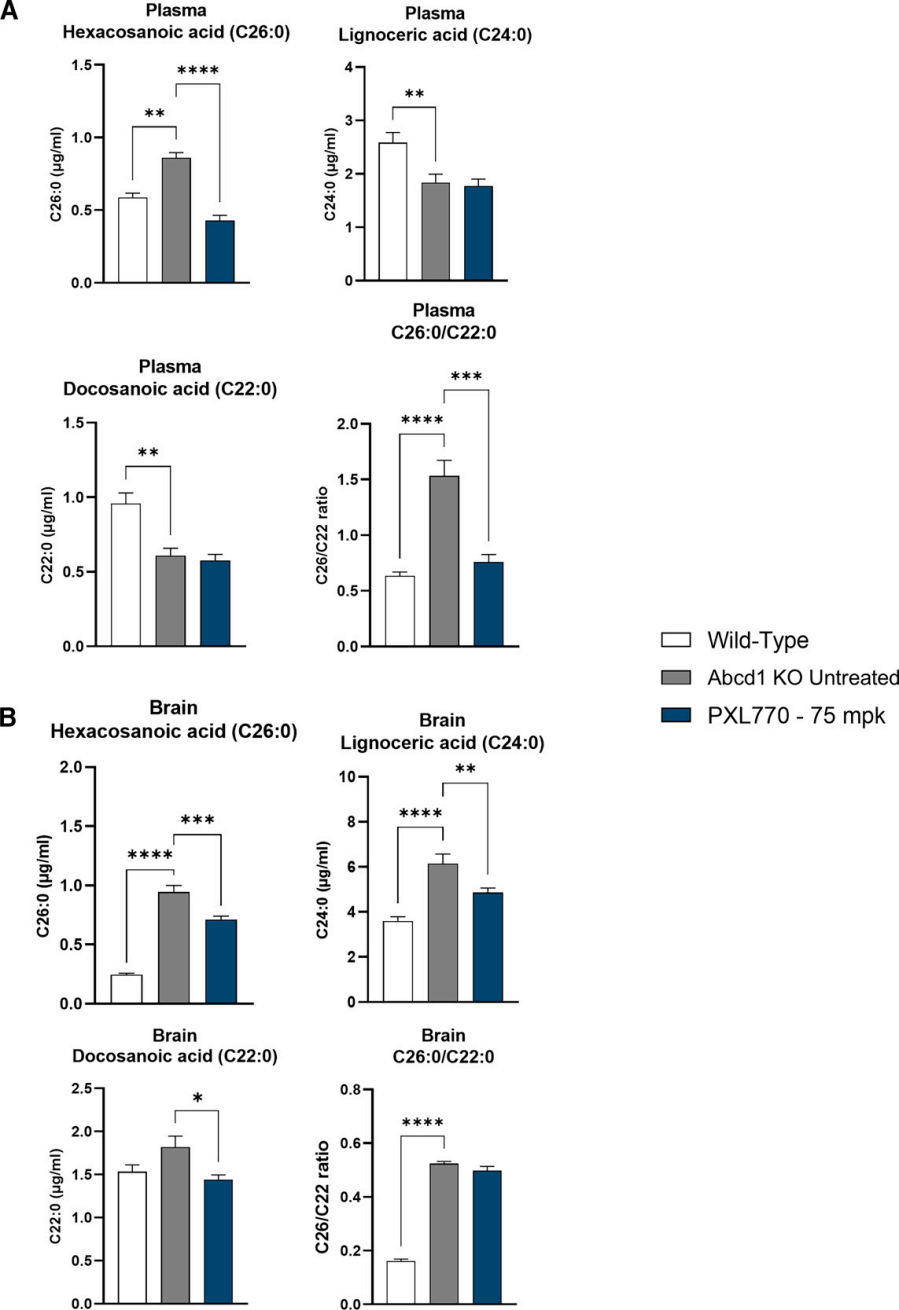
PXL770 improves VLCFA levels in Abcd1 KO mice. VLCFA content measured by LC-MS in (A) plasma and (B) brain from 6- to 8-week-old *Abcd1* KO mice treated with PXL770 at 75 mg/kg (BID – Oral) for 8 weeks. Data are mean ± SEM, *n* = 12 animals for wild-type and *n* = 15 for KO untreated and PXL770. *P < 0.05, **P < 0.01, ***P < 0.001, ****P < 0.0001 (Dunnett’s test versus untreated).

VLCFA accumulation, in particular C26:0, has been described in ALD patients in plasma and key tissues such as brain and spinal cord (Igarashi et al., 1976; Moser, 1997; Engelen et al., 2012; Rattay et al., 2020); similar perturbations are also observed in *Abcd1* KO mice (Pujol et al., 2004). To assess its potential efficacy on ALD in vivo, 6–8-week-old male *Abcd1* KO mice were dosed orally twice daily with PXL770 at 75 mg/kg for 8 weeks (*n* = 12 to 15 animals/condition). In the plasma of *Abcd1* KO mice, C26:0 levels were increased by 46% compared with wt controls (*P* < 0.01, Fig. 5A). As mentioned, an increase in the plasma C26:0/C22:0 ratio is a robust marker for ALD; the ratio was significantly increased by 2.4-fold in *Abcd1* KO mice (*P* < 0.0001 versus wt, Fig. 5A). In line with our in vitro results, PXL770 treatment restored plasma C26:0 levels to normal wt levels in ALD mice compared with untreated (*P* < 0.0001, Fig. 5A). Plasma C24:0 and C22:0 mean levels were unexpectedly lower in untreated ALD mice and remained unchanged after treatment with PXL770 (Fig. 5A). A significant improvement in C26:0/C22:0 ratio by PXL770 treatment was noted and can be attributed to the decrease in C26:0 (*P* < 0.001, Fig. 5A).

In the brain of *Abcd1* KO animals, C26:0 and C24:0 levels were significantly increased, by 3.9- and 1.7-fold compared with wt, respectively (*P* < 0.0001, Fig. 5B), whereas C22:0 levels remained unchanged (Fig. 5B); the C26:0/C22:0 ratio was significantly increased by 3.3-fold (*P* < 0.0001, Fig. 5B). Treatment of ALD mice with PXL770 reduced brain C26:0 and C24:0 in disease mice by 25 and 21%, respectively, compared with untreated ALD mice (C26:0 *P* < 0.001 and C24:0 *P* < 0.01, Fig. 5B). Of note, the treatment had some effect to lower C22:0 as well; this may explain why the C26:0/C22:0 ratio was unaffected by PXL770 treatment in brain from ALD mice (Fig. 5B).

**Fig. 6.**
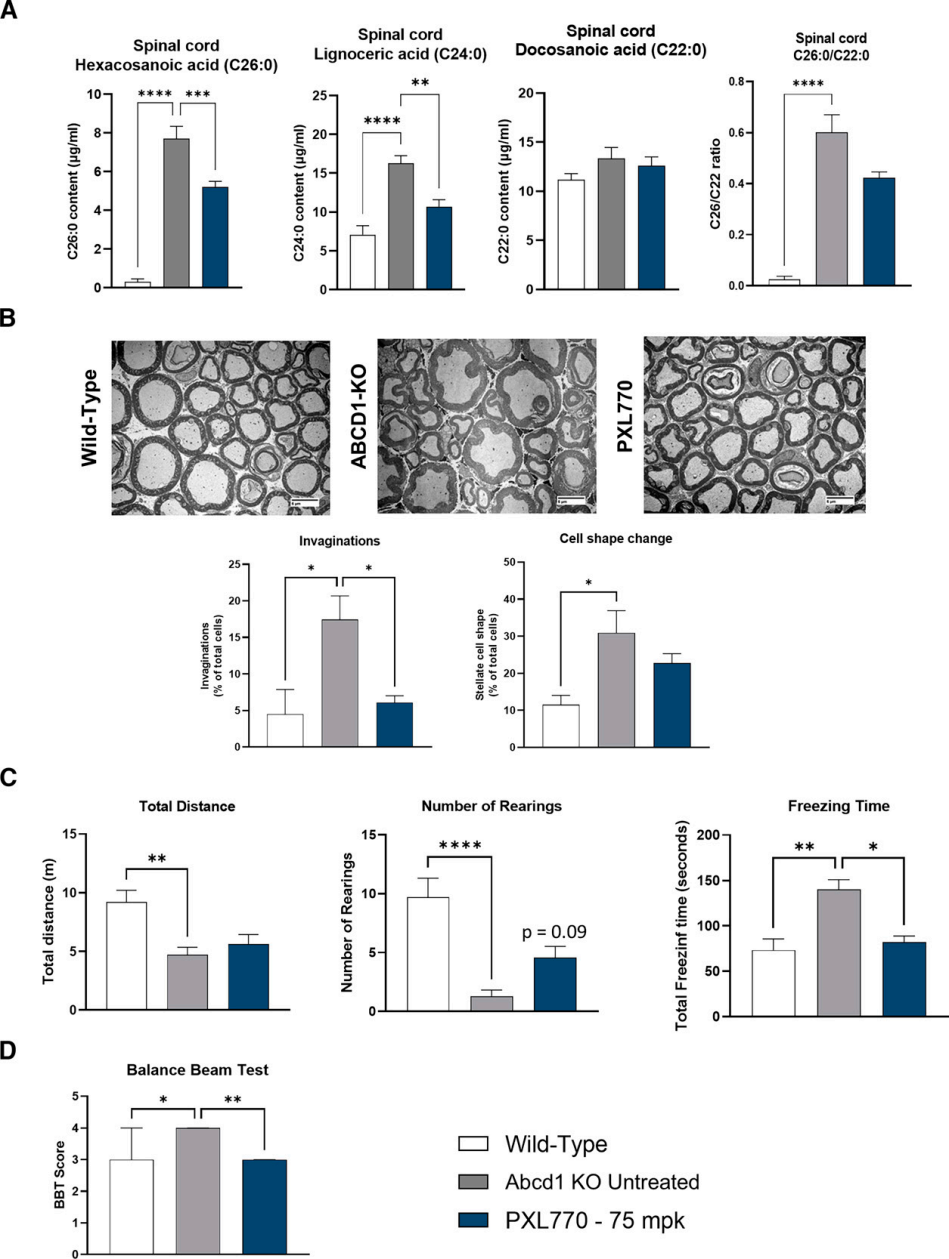
PXL770 treatment improves VLCFA levels in spinal cord, axonal morphology of sciatic nerve and locomotor function in Abcd1 KO mice. 13-month-old Abcd1 KO mice were treated with PXL770 75 mg/kg (BID oral) for 12 weeks. (A) VLCFA content measured by LC-MS in spinal cord. Data are mean ± SEM, *n* = 8 animals/condition. (B) Axonal morphology of myelin and neurons determined by morphometric analysis of transversal slices of the sciatic nerve by EM (magnification 800X). Data are mean ± SEM, *n* = 4 animals per condition. Behavioral and locomotor function assessment by (C) open field test and (D) Balance beam test. Data are median ± 95% interval confidence, *n* = 8 animals for wild-type and KO untreated, 7 animals for PXL770. *P < 0.05, **P < 0.01, ***P < 0.001 ****P < 0.0001 (Dunnett’s test versus untreated).

The *Abcd1* KO mouse only manifests a mild neurologic phenotype beginning with older age – greater than 6 months (Pujol et al., 2002). To further assess potential effects of PXL770 in mice exhibiting a neurologic phenotype, we conducted a separate study where 13-month-old male *Abcd1* KO mice were treated with the compound for 12 weeks (*n* = 8 animals/condition). In this context, we also focused on examining spinal cord tissue. Relative to wt control mice, untreated ALD mice were observed to have an increase in C26:0 and C24:0 (24.8- and 2.3-fold, *P* < 0.0001 versus wt, respectively, Fig. 6A) in this tissue; spinal cord C22:0 remained stable and the C26:0/C22:0 ratio was increased as expected (20-fold, *P* < 0.0001 versus wt, Fig. 6A). In these older mice, PXL770 treatment reduced C26:0 and C24:0 levels in spinal cord compared with untreated ALD animals (-32% and -34%, *P* < 0.001 and *P* < 0.01, respectively, Fig. 6A); there was a trend toward an improvement in C26:0/C22:0 as well.

Collectively, these results indicated that PXL770 treatment in ALD mice decreases VLCFA levels in plasma, brain and in spinal cord, a key tissue in the AMN subtype of ALD.

### PXL770 Treatment Improves Neuronal Morphology and Locomotor Function in *Abcd1* KO Mice

We also performed neuronal morphologic analysis and locomotor functional tests in 13-month-old mice.

Invaginations of the myelin sheath and other morphologic changes in axon shape have been implicated as an early focal defect indicative myelopathy that leads to axonal degeneration (Giese et al., 1992; Pujol et al., 2002; Beirowski et al., 2004). Assessment of axonal morphology by electronic microscopy using axial slices of *Abcd1* KO mice sciatic nerve was performed (*n* = 4 animals/condition); here we observed a significant increase in the proportion of axons with myelin invaginations and “stellate” shape compared with wt (3.9- and 2.7-fold, respectively, *P* < 0.05, Fig. 6B). Treatment with PXL770 allowed for rescue of the myelin invagination finding in *Abcd1* KO mice – reducing mean abnormal cell counts to wt levels (*P* < 0.05 versus untreated, Fig. 6B); as well as producing a trend toward an improvement in mean cell shape (-26%, not significant, Fig. 6B).

Functional neurologic tests – open field and balance beam - as described in Material and Methods – were assessed in 13-month-old ALD animals treated chronically (12 weeks) with PXL770 (*n* = 6 to 8 animals/condition). During the open field test, compared with wt, the total distance traveled by untreated ALD mice was reduced by 49% (*P* < 0.01, Fig. 6C), the number of rearings decreased by 87% (*P* > 0.0001 versus wt [1 outlier value excluded by Graph Pad prism in wt group], Fig. 6C), and mean freezing time was increased by 91% (*P* > 0.01 versus wt, Fig. 6C); these findings were consistent with reduced overall motor function and mobility. Paralleling the improvement in axonal morphology, PXL770 treatment produced a trend to improve the number of rearings (3.6-fold, *P* = 0.09 versus untreated [1 outlier value excluded by Graph Pad prism in untreated group], Fig. 6C) and also restored mean freezing time values down to wt levels (*P* < 0.05 versus untreated [1 outlier value excluded by Graph Pad prism in PXL770 treated group], Fig. 6C). During the balance beam test, the ALD mice showed an increased average score of 4 compared with 3 in the wt mice (*P* < 0.05, Fig. 6D) demonstrating impaired performance; in PXL770 treated mice, the mean score was restored to normal (*P* < 0.01 versus untreated, Fig. 6D).

Altogether, these results indicate that direct AMPK activation mediated by PXL770 in 13-month-old ALD mice with associated clinical phenotypes improved sciatic nerve axonal morphology, along with amelioration of defective locomotor function.

## Discussion

Patients with each of the major overlapping forms of ALD are afflicted by serious, debilitating, and life-threatening symptoms and consequences. Among only a small handful of drug targets including activation of thyroid receptor *β* (Hartley et al., 2017), AMPK has emerged as a possible treatment of ALD (Weidling and Swerdlow, 2016). Suprapharmacologic doses of metformin - a weak and indirect AMPK activator (Singh and Giri, 2014; Singh et al., 2015, 2016) with AMPK-independent effects (Zhou et al., 2001) - modestly lowers VLCFA, improves mitochondrial function, and induces *ABCD2* expression in patient derived cells (Hundal et al., 2000; Miller et al., 2013; Singh et al., 2016). At clinical concentrations, the mechanism of action of metformin differs from suprapharmacologic doses (Wang et al., 2019). It was therefore critically important to address the potential therapeutic benefits of a selective and direct allosteric activator in ALD. In contrast to metformin, the effects of PXL770 were more potent and robust.

VLCFA increases, in particular of C26:0 and the C26:0/C22:0 ratio, are the key hallmark and a proven proximal root cause of ALD (Engelen et al., 2012; Rattay et al., 2020). VLCFA accumulation has been previously reported in skin fibroblasts, various tissues and plasma from ALD patients, and in *Abcd1* KO ALD mice models (Pujol et al., 2002; Kemp et al., 2016). In this study, we confirmed the presence of VLCFA accumulation in all our models including C-ALD and AMN fibroblasts, *Abcd1* KO glial cells, and ALD mouse tissues. Importantly, VLCFA levels were suppressed in every context studied by treatment with PXL770. Prior studies have shown that the VLCFA profile observed in ALD plasma and fibroblasts were not paralleled in lymphocytes (Weber et al., 2014), however we observed similar increases of VLCFA in ALD-derived lymphocytes, which were similarly normalized by PXL770 treatment. In their study (Weber et al., 2014), Weber and colleagues observed higher *ABCD2* expression in lymphocytes and hypothesize that this compensatory mechanism may prevent VLCFA accumulation. In the present study, we observed higher *ABCD2* mRNA levels in lymphocytes from AMN patients versus healthy subjects (even if it does not reach significance); however, under conditions we studied, this small increase was not sufficient to prevent VLCFA accumulation. The substantial induction of the expression of *ABCD2* and *ABCD3* that we observed with PXL770 leads us to speculate that this effect contributes to VLCFA lowering given that the overlapping function of these two transporters with ABCD1, in particular for ABCD2, has been implicated as a potential therapeutic approach (Pujol et al., 2004). However, VLCFA levels were impacted by PXL770 at all tested concentrations in ALD patients’ cells whereas increases in *ABCD2* and *ABCD3* were only observed at the top concentrations; this suggests that other mechanisms are involved in the actions of AMPK to modulate VLCFA homeostasis.

Importantly, we sought to establish if PXL770 treatment could reduce VLCFA in key target tissues in vivo. Of note, a single dose study showed that plasma PK parameters measured in the *Abcd1* null mice were also within, or modestly below, exposure levels previously reported in a Phase 1B study where patients with nonalcoholic fatty liver were receiving 500 mg QD PXL770 (Fouqueray et al., 2021). Although PXL770 in vivo treatment did not fully normalize elevations in VLCFA in brain or spinal cord, significant reductions were noted. Partial correction of elevated tissue VLCFA alone may not be sufficient to result in a clinical benefit; however, we hypothesize that other effects of AMPK activation could impact pathways downstream of VLCFA per se in the mouse disease model – such as mitochondrial function and inflammation as discussed below. This led us to conduct an assessment of histologic and motor outcomes.

Mitochondrial dysfunction and reduced content have been shown to represent major aspects of cellular dysfunction in ALD patients' fibroblasts and tissues as well as in ALD mouse models (Morató et al., 2013; Fourcade et al., 2014), arising before clinical signs occur in ALD mice (López-Erauskin et al., 2013). Moreover, an “energy crisis” caused by defects in multiple glycolytic and TCA cycle-associated enzymes has been documented in *Abcd1* KO mice and in ALD fibroblasts (Galino et al., 2011). Mitochondrial impairment could be a direct consequence of VLCFA accumulation given that C26:0 has established toxic effects on oxidative phosphorylation (López-Erauskin et al., 2013) and promotes opening of the permeability transition pore (Hein et al., 2008). Interestingly, McGuinness observed that peroxisomal VLCFA *β*-oxidation in human and mouse fibroblasts is not solely controlled by ABCD1 function, but also by the rate of mitochondrial long-chain fatty acid *β*-oxidation (McGuinness et al., 2003). This suggests that mitochondrial dysfunction could be an early event participating directly in VLCFA accumulation in the pathophysiology of the disease. Given that AMPK activation can augment mitochondrial function and biogenesis in several other contexts (Herzig and Shaw, 2018), we probed this key potential for therapeutic effect. In the present study we observed a reduction in basal and ATP linked respiration as well as maximal oxidative capacity in C-ALD fibroblasts, consistent with previous findings (Singh and Giri, 2014; Singh et al., 2015). These defects were not readily apparent in cells from AMN patients or glial cells from ALD mice. Nevertheless, PXL770 was shown to augment mitochondrial function in each disease-related cell type we interrogated, supporting potential interest for AMPK activation in improving this component of disease pathology.

Although the precise mechanisms linking VLCFA to ALD phenotypes are still unclear, accumulating evidence suggests that neuroinflammation is a key secondary component of pathophysiology (Singh and Pujol, 2010; Marchetti et al., 2018). In particular, evidence indicates that the transition to rapidly progressive demyelination in C-ALD is mediated by inflammation, including infiltration of mononuclear cells – mostly macrophages – and high levels of inflammatory chemokines – including MCP-1 (Berger et al., 2014). We also previously reported that direct AMPK activation (via PXL770 treatment) has anti-inflammatory effects: including reducing total liver leukocytes - monocytes and resident macrophages – and suppressing MCP-1 levels in a diet-induced obese NASH mouse model, as well as inhibiting proinflammatory cytokine production in human macrophages (Gluais-Dagorn et al., 2022). In this study, we documented a proinflammatory gene expression signature in C-ALD lymphocytes and cytokine stimulated *Abcd1* KO glial cells. Exposure to PXL770 was then shown to exert a potent inhibitory effect on several neuroinflammatory marker genes. This suggests that AMPK activation may not only act on the proximal cause of ALD, but could also reduce the downstream development of inflammation in the disease as well.

After chronic treatment of older (13-month-old) diseased ALD mice, PXL770 was also shown to improve sciatic nerve axon morphology and locomotor function. Older ALD mice develop neurologic disease, including neuronal cell and functional abnormalities, as previously described (Pujol et al., 2002) and confirmed in the present study. Although subtle, the morphologic defects of the myelin sheath and axons of the sciatic nerve – potential early signs of myelopathy and axonal degeneration (Giese et al., 1992; Pujol et al., 2002; Beirowski et al., 2004), are overlapping with selected histopathological observations previously reported in peripheral nerve from patients with AMN (Powers, 1985; Powers et al., 2000). The open field test - measuring general activity and mobility linked to locomotor function (Gould et al., 2009) - in tandem with a balance beam test - assessing motor balance and coordination - allowed us to gauge the potential therapeutic utility of AMPK activation on potentially relevant clinical phenotypes. Substantial and statistically significant efficacy was observed for multiple parameters.

There are several limitations of these experiments. Firstly, a few of the experiments reported here (Fig. 3 in particular) included a small sample size which may have limited statistical power. Secondly, the extent of PXL770 brain penetration has not been characterized. Therefore, it remains uncertain whether effects on axonal morphology and neurologic function are related to – and require - direct compound exposure or not. Additional experiments will be needed to better understand this and the mechanisms responsible for the observed efficacy. In addition, our results did not allow for an assessment of potential correlations between VLCFA accumulation and neuronal efficacy. Additional VLCFA level measurements in brain and plasma of 13-month-old ALD mice would be required to provide a complete picture of efficacy in relation to disease biomarkers. However, plasma VLCFA levels do not correlate with disease severity or progression in humans with ALD, suggesting that a threshold for elevated VLCFA is operative in pathophysiology.

In conclusion, in vitro and in vivo results reported here suggest that direct AMPK activation can positively impact several key molecular and cellular components involved in ALD pathophysiology, such as VLCFA accumulation, inflammation and mitochondrial dysfunction. In addition, positive outcomes observed in the in vivo functional tests suggest that long term clinical administration of PXL770 to patients could yield beneficial effects on motor function. Even though further studies are required to overcome the limitations described above, based upon these positive preclinical results, we plan to initiate clinical development of PXL770 in ALD, beginning with a Phase 2a study in adult male AMN patients to assess its effects over 12 weeks of treatment (ClinicalTrials.gov NCT05146284).

## Abbreviations

ABCD1: ATP binding cassette subfamily D member 1
ALD: X-linked adrenoleukodystrophy
AMN: adrenomyeloneuropathy
AMPK: AMP-activated protein kinase
C-ALD: cerebral ALD
HFHS: Henry Ford Health System
MOC: maximal oxidative capacity
NASH: nonalcoholic steatohepatitis
OCR: oxygen consumption rate
VLCFA: very long-chain fatty acid
